# Clinical Impact of Appendiceal Morphology on Surgical Outcomes and Readmissions: Does Size Matter?

**DOI:** 10.3390/jcm14165635

**Published:** 2025-08-09

**Authors:** Miri Elgabsi, Gal Malkiely, Tal Weiss, Neev Tchernin, Boris Kessel, Veacheslav Zilbermints

**Affiliations:** 1 Division of Surgery, Hillel Yaffe Medical Center, Hadera 3820302, Israel; miriv42@gmail.com (M.E.); magertal@gmail.com (T.W.); neevtch@gmail.com (N.T.); veacheslavz@hymc.gov.il (V.Z.); 2 Rappaport Faculty of Medicine, Technion-Israel Institute of Technology, Haifa 3200003, Israel

**Keywords:** appendicitis, morphology, appendix diameter, appendix length, appendectomy, surgical outcome

## Abstract

**Background:** While the severity of acute appendicitis is routinely evaluated, the significance of its morphological characteristics remains underexplored. This study aimed to evaluate the clinical impacts of appendiceal dimensions. **Methods:** This retrospective study included patients who underwent appendectomy. Data on demographics, appendiceal morphology, time from admission to surgery, postoperative complications, and readmission rates were analyzed. Statistical tests, including correlation analysis and multivariate regression, were used. *p*-value ≤ 0.05 was considered statistically significant. **Results:** Appendix diameter demonstrated positive correlations with age, complicated appendicitis, and surgery duration. Multivariate analysis showed that appendix diameter was found to be a significant predictor of readmission rates, regardless of clinical factors, and has a significant positive association with age in both univariate/multivariate analyses. **Conclusions:** Our findings demonstrate the significance of appendicular morphology in the prediction of readmission rates and the importance of age-specific diagnostic thresholds. The observed age-related changes may warrant re-evaluation of recent diagnostic criteria.

## 1. Introduction

Acute appendicitis (AA) is the most common cause of acute abdominal surgical emergencies requiring surgical intervention [1]. Appendicular morphology varies significantly, with lengths ranging from 0.5 to 23 and an average between 5.3 and 11.7 cm. The normal appendiceal diameter is generally accepted as 3–10 mm, and its enlargement usually indicates pathology [2,3].

The primary pathophysiological mechanism of AA is luminal obstruction, often caused by fecaliths, lymphoid hyperplasia, or other blockages [4]. This obstruction results in bacterial overgrowth and increased mucus secretion, causing elevated intraluminal pressure, ischemia, and, if untreated, eventual necrosis or perforation [5].

The diagnosis often relies on a combination of patient anamnesis, physical examination, laboratory tests, and advanced imaging modalities such as ultrasound and abdominal computed tomography (CT), particularly for atypical presentations [6].

CT imaging has established an average appendiceal diameter of 6.6 mm ± 1.5 mm, with wall thickness rarely exceeding 6 mm [7]. These thresholds are essential for identifying appendicitis, as age-related changes in normal appendix dimensions are minimal. This consistency underscores the utility of fixed diagnostic thresholds. Currently, despite the development of multiple clinical scores and diagnostic techniques, the morphological features of the appendix (length/diameter) remain under-evaluated as markers for both diagnosis and outcomes. While the appendiceal diameter typically increases during inflammation, its length remains relatively constant. Consequently, the length-to-diameter ratio has been proposed as a potential indicator of disease severity [8]. There are multiple factors that may have an influence on appendix diameter, such as anatomical position, timing from the debut of the inflammatory process, and the speed of its progression. However, at the time of the diagnosis, larger diameters and thicker walls are associated with an increased risk of perforation, regardless of its topography [9]. Additionally, a length-to-diameter ratio below 10 has been proposed as an early diagnostic marker for perforation [10].

This study aims to evaluate the role of appendiceal morphology, specifically its length and diameter, in diagnosis, surgical outcomes, and clinical management.

## 2. Patients and Methods

This retrospective, single-center study was conducted at Hillel Yaffe Medical Center, Israel. The study included patients aged 18 years and older who underwent appendectomy due to acute appendicitis between 1 August 2020 and 30 October 2021. Inclusion criteria required documented morphological measurements of the appendix (length and diameter) obtained through pathological examination. Patients with incomplete clinical or pathological data were excluded from the final analysis. The severity of the inflammation, classified as either acute simple or complicated appendicitis, was validated based on the final pathological report.

The study was approved by the local ethics committee (protocol number 0118-23-HYMC).

### 2.1. Data Collection

The data was retrieved retrospectively from hospital electronic medical records. Data collection included patient demographics (age and gender), the time from emergency department admission to surgery, and surgical details: type of procedure (laparoscopic or open), and the method of appendix stump closure (Endoloop, Stapler, or Suturing). Additional clinical data included the duration of the operation (in minutes), the length of hospitalization (in days), and readmission rates, which were defined as hospitalization within one year following appendectomy due to surgery-related complaints. Surgical site infections were classified as superficial (involving only the skin and subcutaneous tissue) or deep (involving the fascial and muscle layers). Morphological measurements, including appendiceal length and diameter, along with pathological diagnoses, were obtained from pathology reports. Pathologists measured the appendix length from the base to the tip using a calibrated ruler. While the appendiceal diameter measured in pathology provides valuable insights into postoperative outcomes, its utility for real-time clinical decision-making is limited as it becomes available only after surgery. To address this limitation, we evaluated preoperative imaging measurements (CT or ultrasound) of appendiceal diameter to assess their correlation with pathological findings. In cases where two radiologic modalities were performed, the diameter of the appendix was confirmed by CT. All radiologic measurements were extracted from electronic reports.

### 2.2. Statistical Analysis

Continuous variables were assessed for normality using the Shapiro–Wilk test. Relationships between appendiceal morphology (length and diameter) and quantitative variables were evaluated using Spearman’s correlation coefficients, appropriate for non-parametric data. Group comparisons of continuous variables were conducted using the Mann–Whitney U test for two groups or the Kruskal–Wallis test for multiple groups. Categorical variables were analyzed using the Pearson χ^2^ test or Fisher’s exact test, as appropriate. Univariate analyses identified potential predictors of surgical outcomes, including operation duration, length of hospitalization, and readmission rates. Significant variables from the univariate analysis were included in multivariate regression models to evaluate their independent effects. All analyses were performed in Python using Pandas and statistical libraries, with statistical significance defined as *p* ≤ 0.05.

## 3. Results

A total of 6 patients were excluded from the study due to incomplete data, resulting in a final cohort of 248 patients, including 123 males (51.7%) and 115 females (48.3%), with a mean age of 38.8 years (range: 18–87 years). The mean appendiceal diameter was 0.99 cm (range: 0.4–2.5 cm).

Imaging was performed using either CT (n = 196, 79.0%) or abdominal ultrasound (US). The mean diameter measured on CT was 1.01 cm (median: 1.00 cm; range: 0.40–2.50 cm), while US imaging showed a mean diameter of 0.96 cm (median: 1.00 cm; range: 0.50–1.50 cm). The mean appendiceal length was 6.57 cm (range: 3–14 cm). The mean time since admission to surgery was 742.04 min (range: 210.12–3512.07 min). Operation duration had a mean of 54.42 min (range: 17–214 min). Most procedures were performed laparoscopically (97.5%), while open surgeries accounted for only 2.52% of cases. Closure methods for the appendix stump were predominantly Endoloop (87.4%), followed by Suturing (6.7%) and Stapler (5.9%).

Pathological diagnosis reported that Acute Simple Appendicitis comprised 81.1% of cases, with a mean diameter of 0.98 cm. Complicated Appendicitis, including gangrenous and perforated appendicitis with or without peritonitis, accounted for 18.5% of cases and had a mean diameter of 1.09 cm. Appendix Tumor was observed in 0.4% of cases and had a mean diameter of 0.80 cm.

Readmission occurred in 4.2% of cases, with a mean interval to readmission of 52 days (range: 3–292 days). The most common reason for readmission was deep site surgical infection (four cases), all of them treated with antibiotics alone. One patient was readmitted due to a superficial surgical site infection. Three others presented with abdominal pain and vomiting without signs of bowel obstruction. One patient experienced abdominal pain accompanied by postoperative changes observed on imaging without collection. One patient was hospitalized for surgical repair of an incisional port-site hernia, who had the longest interval to readmission, occurring 292 days post-surgery.

Appendix diameter was positively correlated with age (r = 0.132, *p* = 0.042) and operation duration (r = 0.215, *p* = 0.001).

Additionally, the appendix diameter showed a significant negative correlation with time to surgery (r = −0.196, *p* = 0.002). Appendix length demonstrated limited associations, with only a weak but significant positive correlation with operation duration (r = 0.159, *p* = 0.014) (Table 1).

Male patients had a significantly longer appendix length compared to females (*p* = 0.007), with a mean length of 6.9 cm in males versus 6.3 cm in females. There was no difference in diameter found between both genders (*p* = 0.849). A significant association was identified between appendiceal diameter and readmission rates (*p* = 0.004), with larger diameters observed in patients who experienced readmissions. No significant associations were observed between appendiceal length and readmission rates (*p* = 0.129) or between appendiceal morphology and type of surgical procedure (laparoscopic vs. open) (Table 2).

A significant relationship was identified between closure methods of appendicular stump and appendix diameter (H = 14.805, *p* = 0.001). The Stapler group exhibited the largest mean diameter (1.22 cm), followed by the Suturing group (0.98 cm) and the Endoloop group (0.98 cm). Post-hoc analysis revealed significant differences in diameter between the Stapler and Endoloop groups (*p* = 0.000) and between the Stapler and Suturing groups (*p* = 0.011).

In terms of operation duration, Stapler used was associated with longer durations (*p* = 0.001) (Table 2).

Significant differences in appendiceal length were observed between cases of acute simple appendicitis and complicated appendicitis (H = 9.769, *p* = 0.008). The mean length of the appendix was 7.38 cm in cases of acute simple appendicitis, compared to 6.51 cm in cases of complicated appendicitis (*p* = 0.005). No significant differences were found involving the tumor group.

For appendiceal diameter, even stronger differences were identified (H = 21.587, *p* < 0.001). Complicated Appendicitis exhibited a significantly larger mean diameter (1.09 cm) compared to Acute Simple Appendicitis (0.98 cm), *p* < 0.001. The tumor group had a mean diameter of 0.80 cm, though no significant differences were observed between this group and the others, likely due to the small sample size.

### Multivariate Analysis

Multivariate regression analysis revealed that appendix diameter was significantly associated with readmission rates (*p* = 0.001), despite the limited number of readmission cases (n = 10), indicating that a wider appendix diameter increases the probability of readmissions (Table 3).

However, neither appendix diameter (*p* = 0.184) nor appendix length (*p* = 0.161) was found to be statistically associated with operation duration. Similarly, neither diameter (*p* = 0.383) nor length (*p* = 0.735) was significantly associated with length of hospitalization.

Notably, in contrast to the univariate model and clinical expectations, pathology diagnosis (acute vs. complicated appendicitis) was not significantly associated with readmissions. Similarly, the time interval between admission to the emergency department and surgery was not a significant predictor of readmissions. Furthermore, no significant associations were observed between these variables and operation duration or length of hospitalization, emphasizing the need to explore appendix diameter and other clinical variables beyond pathology diagnosis and time to surgery.

Age emerged as the most consistent and significant predictor in the multivariate model. Age was strongly associated with operation duration (*p* < 0.001) and length of hospitalization (*p* = 0.027). Additionally, operation duration itself was significantly associated with length of hospitalization (*p* = 0.002).

A significant positive association was observed between appendix diameter and patient age (r = 0.132, *p* = 0.042). This association remained significant in the multivariate model after adjusting for confounding factors such as operation duration, time to surgery, and pathological diagnosis (*p* = 0.044).

**Figure 1:**
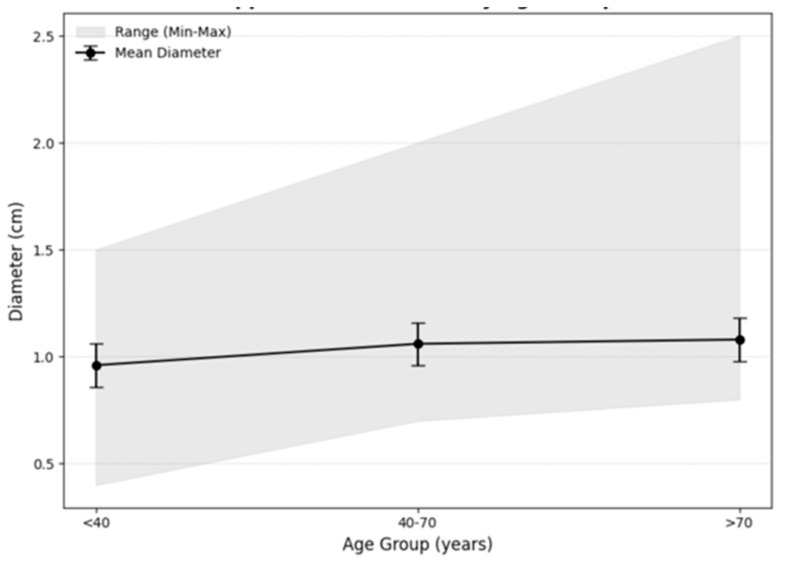
Appendiceal diameter by age groups.

In order to evaluate the relationship between age and appendix diameter, the patients were divided into three age groups: under 40 years, 40–70 years, and over 70 years. The mean diameter increased across groups, from 0.96 cm (range: 0.4–2.5 cm) in patients under 40, to 1.06 cm (range: 0.7–2.0 cm) in the 40–70 age group, and 1.08 cm (range: 0.8–1.5 cm) in those over 70. Significant differences in diameter were observed (H = 9.50, *p* = 0.01), particularly between patients under 40 and those aged 40–70 (*p* = 0.033). No significant difference was observed between the 40–70 and over 70 groups (*p* = 0.086), suggesting a plateau effect in older populations (Figure 1).

## 4. Discussion

This study aimed to examine the relationship between appendix morphology (length and diameter) and clinical variables, as well as operative and postoperative outcomes.

In univariate analysis, significant relationships were observed between appendiceal diameter and clinical variables, including age, operation duration—potentially reflecting greater surgical complexity—and time from admission to surgery. Appendix diameter was also significantly associated with complicated appendicitis pathology. In contrast, appendix length showed limited associations, with a weak but significant correlation to operation duration and gender, with males exhibiting significantly longer appendices than females. These findings are consistent with previous studies that noted minimal impact of gender on diagnostic thresholds in adult populations [11]. Length in our study did not show a significant correlation with perforation risk, aligning with findings from Dibekoğlu, who similarly reported no association between appendiceal length and perforation [12].

Pathological diagnoses demonstrated significant differences in appendiceal diameter and length, with larger diameters and longer operations favoring the complicated appendicitis group. This supports the hypothesis that appendiceal diameter reflects pathological progression, with larger diameters marking advanced inflammatory states.

Additionally, appendix stump closure methods were associated with larger diameters and longer operation durations in the Stapler group.

Multivariate regression analysis revealed that appendix diameter was significantly associated with readmission rates (*p* = 0.001), with wider diameters linked to an increased probability of readmissions. Most readmissions were due to infections, aligning with findings from Liang et al. [13], who demonstrated that a larger appendiceal diameter predicts an increased risk of wound infections following laparoscopic appendectomy. Importantly, this association was independent of other factors, including appendiceal pathology (acute vs. complicated appendicitis) or the time from admission to surgery, highlighting the unique role of diameter in predicting postoperative outcomes.

Age also played a significant role in appendiceal diameter variation. A positive correlation was observed between age and appendiceal diameter in univariate analysis (ρ = 0.132, *p* = 0.042), which remained significant in the multivariate model after adjusting for confounding factors (*p* = 0.044). When age was analyzed categorically, patients aged 40–70 exhibited larger diameters compared to those under 40 (*p* = 0.033), while no significant difference was observed between patients aged 40–70 and those over 70 (*p* = 0.086). The mean appendix diameter increased consistently with age (0.96, 1.06, and 1.08 cm), as did the minimum diameter (0.4, 0.7, and 0.8 cm, respectively), across the age groups under 40, 40–70, and over 70. The observed increase in appendix diameter with age has important diagnostic implications, particularly for older populations. Measurements that might indicate pathology in younger adults (e.g., a diameter of 1.0 cm) could represent normal findings in individuals over 70 years. This trend aligns with changes observed in other tubular anatomical structures, such as the common bile duct (CBD), where diameter increases by approximately 1 mm per decade, reflecting age-related adaptations [14,15]. These findings emphasize the diagnostic importance of accounting for age-related changes in appendiceal diameter.

Contrary to our findings, which identified a relationship between age and appendiceal diameter, several studies have reported no such association. A sonographic study spanning pediatric and adult populations demonstrated that an appendiceal diameter > 6 mm remains a reliable marker for diagnosing appendicitis, independent of age [16]. Similarly, sonographic studies in pediatric populations have shown minimal correlation between age and appendiceal diameter, instead identifying body weight as a more significant predictor of diameter [17]. Neal et al. found that an appendiceal diameter greater than 7 mm was more predictive of appendicitis than the traditional 6 mm threshold, particularly in younger children [18]. Notably, this aligns with our suggestion that the diagnostic threshold for appendiceal diameter should increase in adults, particularly in older patients, to account for physiological changes and reduce the risk of overdiagnosis. Additionally, a study conducted in Bangladeshi adults observed a significant reduction in appendiceal diameter with age, with measurements taken at the base, middle, and tip showing a decrease from 6.50 mm in individuals under 20 years to 5.51 mm in those over 60 years [19].

In recent years, there has been a growing interest in the conservative management of uncomplicated appendicitis. Studies have demonstrated that larger appendiceal diameters are often predictive of treatment failure in nonoperative approaches [20,21]. Given that older patients face increased risks of surgical complications, including infections, prolonged recovery periods, and higher mortality rates [22], tailoring treatment strategies based on appendiceal diameter could provide significant clinical benefits. However, the risk of missed appendiceal cancers in nonoperative management should also be considered, as highlighted by Meier et al., who estimated an incidence of 1.7–3.9% in patients aged 65 years and older [23]. This underscores the need for further research into the relationship between appendiceal diameter and the risk of malignancies [24].

The economic burden of appendectomy, particularly in older patients, is considerable, with costs escalating in cases involving perforation or sepsis [25].

Importantly, a positive correlation was observed between both CT and US measurements and pathology findings (r = 0.573) and (r = 0.148), respectively; (*p* < 0.001), underscoring their utility as surrogates for postoperative pathological evaluations.

### Strengths and Limitations

This study has some limitations, mostly resulting from a retrospective design, the relatively small study population, and being performed in a single center. While multivariate analysis showed a significant association between appendix diameter and readmission rates, the small number of readmission cases (n = 10, 4.2%) necessitates cautious interpretation of these findings. Another limitation is the relatively short period of time of the study—1 year. An additional limitation is potential bias due to variations in experience of medical staff performing the procedures, which were not standardized or controlled. In addition, the database did not capture the time elapsed from the onset of symptoms to hospital arrival.

However, despite these limitations, the present research is among the few studies evaluating the clinical significance of appendicular morphology. Our data incorporated the final diagnosis based on pathology results, which ensures its accuracy.

## 5. Conclusions

Our findings demonstrate that the diameter of the inflamed appendix is significantly associated with key clinical outcomes, including operative duration, surgical complexity, and postoperative infections. Furthermore, a wider appendiceal diameter was found to be significantly correlated with higher readmission rates, whereas no association was observed between appendiceal length and clinical outcomes evaluated in this study. Our results also demonstrated that the diameter of the inflamed appendix increases with age. We believe these findings provide valuable insights into existing knowledge and may warrant further prospective research to establish better age-specific thresholds for acute appendicitis diagnosis accuracy, particularly in older populations.

## Figures and Tables

**Table 1:** Spearman correlations of appendix length and diameter with clinical variables.

Appendix Morphology	Variable	Correlation (r)	*p*-Value
Length (cm)	length of hospitalization (days)	0.033	0.610
	Age (years)	0.042	0.515
	Admission to operation (minutes)	−0.056	0.388
	Operation Duration (minutes)	0.159	0.014
Diameter (cm)	length of hospitalization (days)	−0.021	0.745
	Age (years)	0.132	0.042
	Admission to operation (minutes)	−0.196	0.002
	Operation duration (minutes)	0.215	0.001

**Table 2:** Group Comparisons of Appendix Length and Diameter Among Clinical Variables.

Variable	Comparison Group	U/H *-Value	*p*-Value
Length (cm)	Gender	5645.500	0.007
	Readmission	1461.500	0.129
	Procedure type	571.000	0.451
	Closure methods for appendix stump	1.762	0.414
	Pathology diagnosis group	9.769	0.008
	Age category	2.501	0.286
Diameter (cm)	Gender	6974.000	0.849
	Readmission	1736.000	0.004
	Procedure type	494.500	0.212
	Closure methods for appendix stump	14.805	0.001
	Pathology diagnosis group	21.587	0.000
	Age category	9.498	0.009

* U-Value: Mann–Whitney U test; H-Value: Kruskal–Wallis test, applied as appropriate.

**Table 3:** Multivariate Regression Analysis of Readmission Rates.

Variable	Coefficient	Standard Error	*t*-Statistic	*p*-Value
const	−0.1937	0.085	−2.276	0.024
Appendix Diameter (cm)	0.1699	0.048	3.524	0.001
Appendix Length (cm)	0.0067	0.009	0.754	0.452
Age	−0.0002	0.001	−0.241	0.81
Operation Duration (min)	0.0005	0.001	0.974	0.331
Length Of Hospitalization (Days)	0.008	0.012	0.684	0.495
Admission to Operation (min)	−1 × 10^−5^	3.18 × 10^−5^	−0.319	0.75
Procedure Type (Open/Laparoscopic)	0.1415	0.084	1.682	0.094
Gender (Male/Female)	−0.0044	0.026	−0.168	0.867
Pathology Diagnosis Group	−0.0304	0.034	−0.897	0.37

## Data Availability

Data can be found at the Hillel Yaffe Medical Center archives.
